# Types and Applications of Nicking Enzyme-Combined Isothermal Amplification

**DOI:** 10.3390/ijms23094620

**Published:** 2022-04-21

**Authors:** Siyu Cao, Xiaochen Tang, Tianshu Chen, Guifang Chen

**Affiliations:** 1 Center for Molecular Recognition and Biosensing, School of Life Sciences, Shanghai University, Shanghai 200444, China; siyucao@shu.edu.cn; 2 Department of Clinical Laboratory Medicine, Shanghai Children’s Medical Center, School of Medicine, Shanghai Jiao Tong University, Shanghai 200127, China; txc0710lss@sjtu.edu.cn

**Keywords:** nicking enzyme, isothermal amplification, molecular detection

## Abstract

Due to the sudden outbreak of COVID-19 at the end of 2019, rapid detection has become an urgent need for community clinics and hospitals. The rapid development of isothermal amplification detection technology for nucleic acids in the field of molecular diagnostic point-of-care testing (POCT) has gained a great deal of attention in recent years. Thanks to intensive research on nicking enzymes, nicking enzyme-combined isothermal amplification has become a promising platform for rapid detection. This is a novel technique that uses nicking enzymes to improve ordinary isothermal amplification. It has garnered significant interest as it overcomes the complexity of traditional molecular diagnostics and is not subject to temperature limitations, relying on cleavage enzymes to efficiently amplify targets in a very short time to provide a high level of amplification efficiency. In recent years, several types of nicking enzyme-combined isothermal amplification have been developed and they have shown great potential in molecular diagnosis, immunodiagnosis, biochemical identification, and other fields. However, this kind of amplification has some disadvantages. In this review, the principles, advantages and disadvantages, and applications of several nicking enzyme-combined isothermal amplification techniques are reviewed and the prospects for the development of these techniques are also considered.

## 1. Introduction

Since the development of polymerase chain reaction (PCR) in 1985, nucleic acid amplification has played an increasingly vital role in biological analysis and clinical diagnosis [1]. Currently, there are two main types of nucleic acid amplification. One of them is the temperature change system and the most representative is PCR.

PCR is the most widely used type of amplification [2]. However, there remain several challenging issues, including [3] error amplification [4,5] and sequence mismatch. These drawbacks, combined with the mandatory requirements for thermal cycling conditions, pose serious obstacles to the miniaturization of PCR instruments [6,7]. Thus, a second type of nucleic acid amplification method has been developed, which is called isothermal amplification. Compared with other nucleic acid amplification techniques, isothermal amplification has the advantages of rapidity [8], high efficiency, high specificity, and no need for special equipment [9]. Therefore, it has gained widespread attention in the past several years.

**Figure 1:**
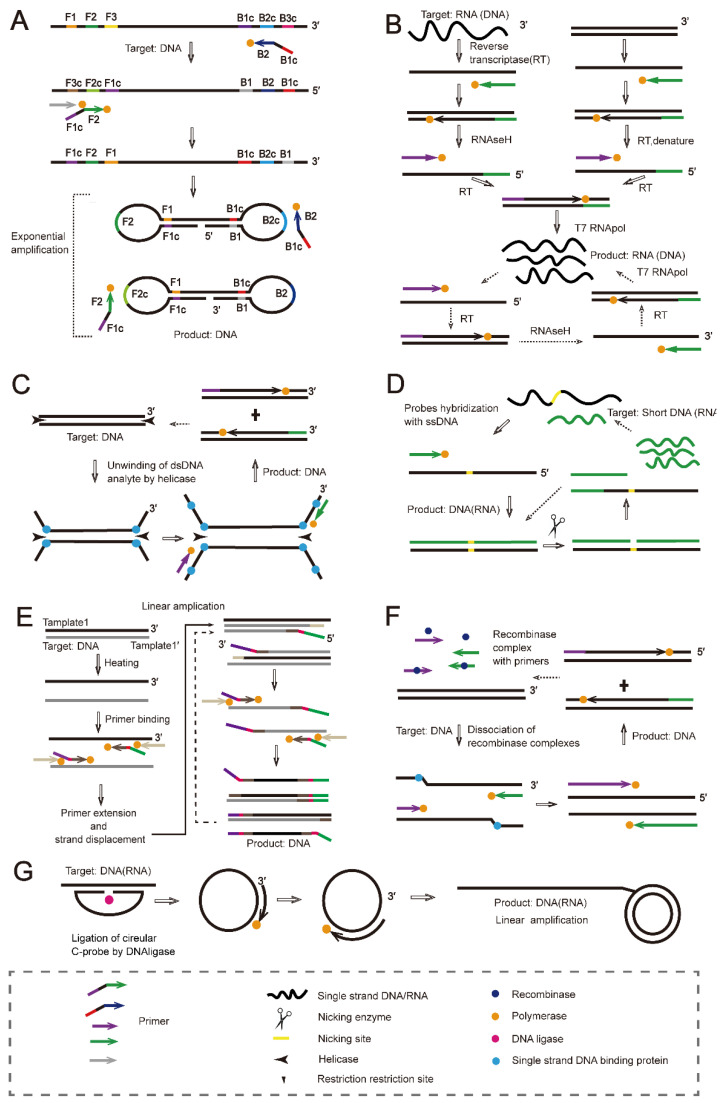
Partial isothermal amplification techniques: (**A**) loop-mediated isothermal DNA amplification (LAMP); (**B**) nucleic acid sequence amplification (NASBA); (**C**) helicase-dependent DNA amplification (HDA); (**D**) exponential amplification reaction (EXPAR); (**E**) linear strand displacement amplification (SDA); (**F**) recombinase polymerase amplification (RPA); (**G**) linear rolling circle amplification (RCA).

Isothermal amplification technologies have been developed in recent years and used to detect bacteria, viruses, fungi, and parasites in clinical [10,11], environmental and food samples [12]. These technologies include: loop-mediated isothermal amplification (LAMP) [13] (Figure 1A), nucleic acid sequence-based amplification (NASBA) [14] (Figure 1B), helicase-dependent amplification (HDA) [15] (Figure 1C), exponential amplification reaction (EXPAR) [16] (Figure 1D), strand displacement amplification (SDA) [17] (Figure 1E), recombinase polymerase amplification (RPA) [18] (Figure 1F), rolling circle amplification (RCA) [19,20] (Figure 1G). Last and most important of all is nicking enzyme-combined isothermal amplification, which is the focus of this review.

**Scheme 1:**
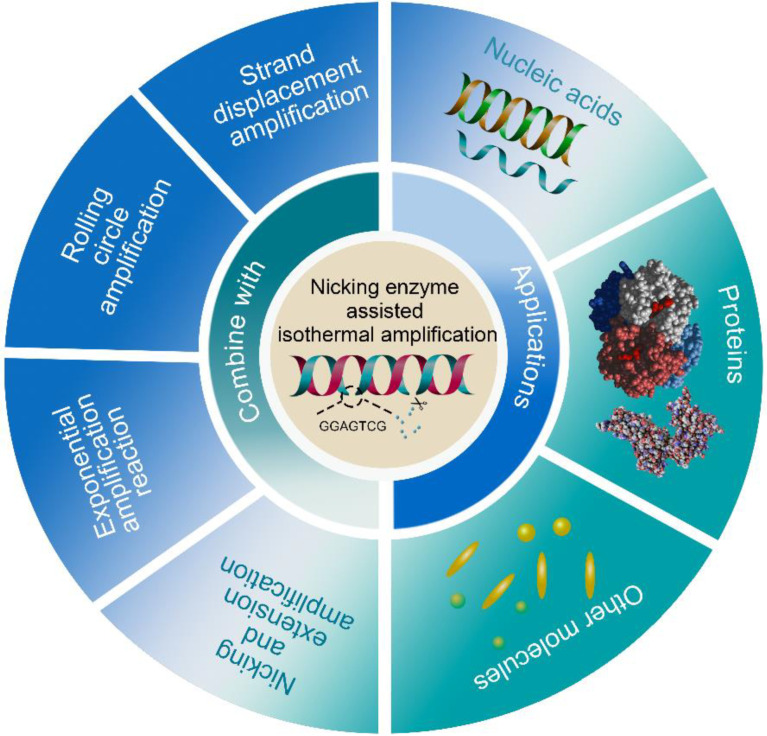
A general summary of four isothermal amplification methods mediated by nicking enzymes, which are widely used in the detection of nucleic acids, proteins, and other small molecules.

In this review, we divide nicking enzyme-combined isothermal amplification into four categories based on the combination of different types of isothermal amplification with nicking enzymes and review the respective applications of these techniques (as shown in Scheme 1).

## 2. Nicking Enzyme-Combined Isothermal Amplification Technology

### 2.1. Nicking Enzymes

In nicking enzyme-combined isothermal amplification technology, the main players are nicking enzymes, also known as nicking endonucleases (NEases) [21,22]. Nicking enzymes are novel enzymes derived from a mutation of restriction enzymes. Like restriction enzymes, they consistently recognize short double strands. The way in which they differ from restriction enzymes is that nicking enzymes are unable to cut the second strand with the loss of dimerization, so they can only break a single strand of a double strand with a particular palindrome sequence [23,24]. This statement is corroborated by L. A. Zheleznaya et al. [21], who reported that nicking enzymes constitute subunits of heterodimeric restriction enzymes [25,26,27].

Most natural nicking enzymes are isolated from a prokaryote called *Bacillus* [28], while some are obtained from viruses [29]. The first nicking enzyme (Nt. CviPII) was discovered in 1988, and then, in 1996, another nicking enzyme, Nt. BstSEI, was discovered. The letters “Nt” or “Nb” indicate whether it is the top (Nt) or bottom (Nb) strand that is cut by the enzyme (previously only the symbol “N” was used) [30]. Li et al. [23] identified and studied the biological functions of about 200 kinds of nicking enzymes in 2011. Table 1 lists the important nicking enzymes and their recognition sequences and isolation source.

### 2.2. Other Components

DNA polymerase [40,41] is also indispensable in nicking enzyme-combined isothermal amplification systems. DNA polymerases with strand displacement activity include Phi 29, Klenow Fragment, vent [42], and Bst DNA polymerase. Among these enzymes, the most commonly used is Bst DNA polymerase [43,44]. Other reactive materials are needed in this system, such as buffer mixture, dNTP, and primers. Once all the materials are present, the two enzymes interact with each other in a suitable system and the target DNA can be amplified exponentially [45,46].

When the above components are present, a suitable temperature is applied, and nicking enzyme-combined amplification begins. Regardless of the type of isothermal amplification, nicking enzyme-combined amplification includes the following four steps: (1) the nicking enzyme recognizes and cleaves a specific site on a single strand in the double strand, exposing the 3′ end; (2) the polymerase extends a new strand from the 3′ end, the new strand containing the recognition site for the nicking enzyme; (3) the nascent strands may be used directly as a product but are more often used to participate in downstream reactions; (4) a large amount of target DNA/RNA product is obtained. This review divides nicking enzyme-combined isothermal amplification into four categories and the specific reaction rules for each category will be described in Section III.

## 3. The Principle of the Mechanism, Derived Types, and Their Applications

As mentioned above, nicking enzymes play the leading role in isothermal amplification technology. Due to its unique cutting site, a nucleic acid strand can be cut at a specific point only with a little design, so it is an indispensable tool in many studies [47,48]. As early as 2015, Chunhai Fan et al. [49] proposed the concepts of Exponential Strand Displacement Amplification (E-SDA), Exponential Rolling Circle Amplification (E-RCA), Linear SDA [50], and Linear RCA. This linear to exponential transformation is attributed to the transformation of the original isothermal amplification technique [51] by nicking enzymes. In this section, we discuss the principles and application examples of several different types of nicking enzyme-combined isothermal amplification methods.

### 3.1. Typical Nicking Enzyme-Combined Amplification: EXPAR

**Figure 2:**
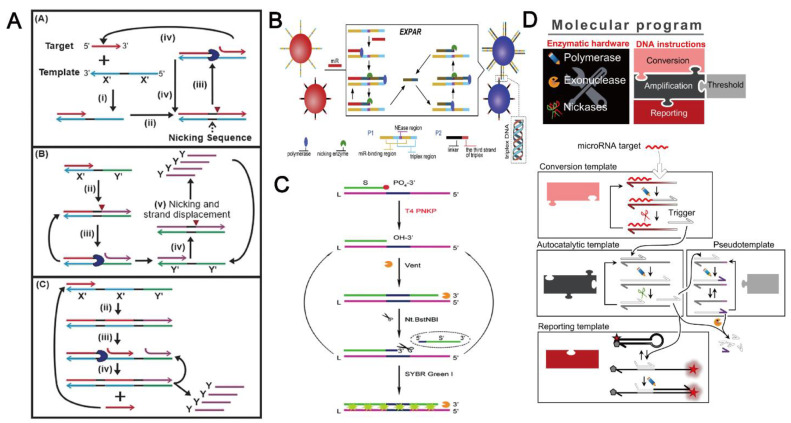
(**A**) Basic principle of EXPAR. From top to bottom: one-template EXPAR system, two-template EXPAR system, and Y’-X’-X’ template system. (Reprinted with permission from Ref. [42]. Copyright 2018 Wiley VCH Verlag GmbH & Co. KGaA, Weinheim.) EXPAR works with nicking enzymes to detect (**B**) miRNA. (Reprinted with permission from Ref. [59]. Copyright 2020 Elsevier.) (**C**) Enzyme activity. (Reprinted with permission from Ref. [60]. Copyright 2019 Royal Society of Chemistry.) (**D**) miRNA. (Reprinted with permission from Ref. [61]. Copyright 2020 Science.).

EXPAR, first reported in 2003 by Galas and co-workers [52], can amplify a signal 10^6^-10^9^ times in a few minutes. This method uses a DNA template, deoxynucleotide triphosphate (dNTPs), and two enzymatic reactions to achieve exponential amplification of a target sequence (Figure 2A). EXPAR uses a functional template (often referred to as an X’-X’ template, where X’ is the complement to the trigger sequence and the middle (–) is the nicking enzyme recognition sequence) with identical fragments at both ends and an intermediate nicking enzyme recognition sequence which can quickly synthesize short oligonucleotides that act as trigger strands to achieve exponential amplification [53]. The trigger sequence, once hybridized with the template, is extended along the template by DNA polymerase with strand displacement activity. The extended dsDNA contains the recognition sequence of the nicking enzyme, which cleaves the dsDNA to create a nicking site. DNA polymerase can extend the 3′ end of the nicking site while displacing the newly synthesized strand. Since the template contains two X’ regions, the displaced DNA strand has the same sequence as the triggering DNA. In order to achieve exponential amplification, the initial target DNA amplification of the triggering–template complex continues, and then a new round of amplification begins with the displaced strand hybridizing with another template.

EXPAR has been applied in many studies [54,55]. MicroRNAs (miRNAs) are a class of non-coding single-stranded RNA molecules about 22 nucleotides in length [56]. Although the length is shorter, they are involved in the regulation of post-transcriptional gene expression in plants, animals, and viruses [57], and some of them can even be used as therapeutic targets [58]. As shown in Figure 2B, Shuangqin Wei et al. [59] used EXPAR technology to detect miRNA. In this study, they established an miRNA detection method based on EXPAR and three-stranded DNA-mediated gold nanoparticle aggregation (AuNPs). The target-triggered aggregation of AuNPs resulted in a significant change in the UV absorption spectrum, and the color of the reaction solution also changed from red to purple, which could be monitored in real time and detected even with the naked eye. Specifically, a class of AuNPs binds to the EXPAR probe, on which complementary sequences of target miRNAs are located. In the presence of a target miRNA, the EXPAR reaction is triggered to form double-stranded DNA on the surface of AuNPs. Then, a single-stranded DNA probe on another class of AuNPs interacts with the double-stranded DNA to form three-stranded DNA, resulting in the collection of two classes of AuNPs, which can be quantified by UV–Vis. The detection limit of this method is 0.23fM, and the entire detection process only takes 30 min. This method is simple, fast, has high selectivity and accuracy, and is expected to meet the needs of real-time detection of miRNA.

Helped by nicking enzymes, EXPAR can also be used to detect enzyme activity. For example, as a bifunctional enzyme, T4 polynucleotide kinase phosphatase (T4 PNKP) not only catalyzes the phosphorylation of the 5′ hydroxyl group but can also remove the terminal 3′ phosphate group. Moreover, T4 PNKP is closely related to the recombination, replication, and damage repair of nucleic acid. Therefore, Huinan Chen et al. [60] constructed a new method based on EXPAR technology to detect the activity of T4 PNKP. As shown in Figure 2C, a strand containing a phosphate group at the 3′ terminus, named S (green), was designed as a substrate for T4PNK. The L strand as the template consists of three parts, the nicking enzyme recognition sequence in the middle (blue) and the same sequence at both ends (pink). At the same time, the 3′ end of the L strand is modified with an amino group to prevent nonspecific amplification. In the presence of T4 PNKP, the 3′ phosphate group at the end of the S strand is cut, resulting in the exposure of the 3′ hydroxyl end. With vent polymerase, the obtained S strand can produce an S’ strand. Subsequently, many S’ strands are released after cutting by Nt. BstNBI and can be used as primers to trigger continuous amplification reactions. Due to the number of products, the resulting strands will form many double strands in the final system. After staining with SYBR Green I, a significant fluorescent signal was produced. Thus, using the EXPAR technique, the researchers succeeded in converting the kinase activity to fluorescent intensity. Coincidentally, in the study of YuPeng Zhang et al., EXPAR was also used to detect the activity of enzymes, indicating the importance of EXPAR technology.

Guillaume Gines et al. [61] invented a molecular program, using an isothermal amplification chemistry adapted to a droplet digital readout to detect miRNA sensitively and quantitatively. As shown in Figure 2D, the molecular program is encoded by four consecutive DNA modules and catalyzed by a set of enzymes; in addition to polymerase and exonuclease, there is a nicking enzyme. The four modules work as follows: first, the conversion template (CT module) converts the target miRNA into a general S-strand sequence and then the autocatalytic template (AT module) index amplifies the S-strand sequence. Then, in order to avoid target-independent amplification, the pseudo-template (PT module) drives the inactivation of a small number of triggers from the leak reaction. Finally, the reporting template (RT module) converts the S-stand into a fluorescence signal. The whole time, in the AT, PT, and RT modules, nicking enzymes are working, playing a good supporting role. The program overcomes the disadvantage of EXPAR’s tendency to produce leakage reactions and avoids the eventual triggering of the production of amplified products from spurious reactions.

### 3.2. Nicking Enzymes Combined with SDA

In 1992, Walker et al. [62,63] published a study on SDA, which marked the birth of a new DNA amplification technique. In 1994, Walker et al. [64] expanded the amplification sequence of SDA into two based on the original single-target SDA (an SDA that amplifies only one target fragment by linear amplification), namely, established multiplex SDA. SDA developed most rapidly in the year 1996, and both the SDA system and the SDA product detection method represented great innovations [65]. The early SDA technique was not simple enough, mainly for the following reasons: for the preparation of a target DNA template, four primers were required to carry out displacement reactions (Figure 1E), or restriction enzyme-mediated cleavage was used (such as HincⅡ and Hind Ⅱ). However, phosphoric acid is usually introduced into the system to prevent a restriction enzyme from cutting into the second strand of the double chain. Both methods are cumbersome. Fortunately, these difficulties have been eliminated using nicking enzymes, which only cut one dsDNA strand.

**Figure 3:**
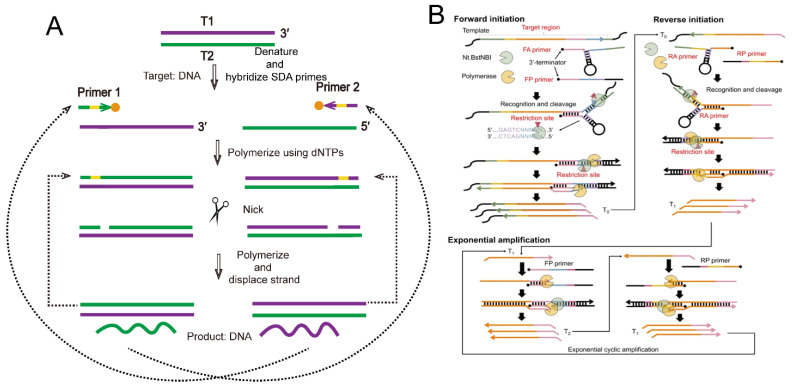
(**A**) Basic principle of nicking enzymes combined with SDA. (**B**) Example of nicking enzyme combined with SDA to detect RNA. (Reprinted with permission from Ref. [66]. Copyright 2021 American Chemical Society.).

The technology is still being developed today. The main mechanism of SDA is based on the continuous nicking and polymerization/displacement process catalyzed by nicking enzymes and DNA polymerases. The specific steps are shown in Figure 3A. In the presence of primer 1, primer 2, template 1, and template 2, two primer–template complexes are formed after denaturation. Both primers contain an identification sequence that is used to cause the identification cleavage of the nicking enzyme. After being cut, DNA polymerase extends the 3′ end of the double strand to generate dsDNA containing the complete nicking site, which will be cut by a nicking enzyme to generate a new 3′ end at the notch, triggering a new extension reaction and causing the displacement of the downstream target strand. The ssDNA from the primer1–template1 complex can be used as a template for primer 2, and the product from the primer2–template2 complex can also be used as template for primer1. This cycle results in exponential amplification of the target.

There are some examples of SDA working with nicking enzymes [67], such as RNA amplification and detection [68]. Chao Zhang et al. [66] reported a simple and versatile method for detecting RNA based on an aligner-mediated cleavage-based strand displacement amplification (AMC-SDA) reaction. In this study, a nicking enzyme and SDA were used together to perform a simple and general measurement of SARS-CoV-2 RNA. The scheme involves three steps: forward priming, reverse priming, and exponential amplification (Figure 3B). First, the target DNA template is hybridized with a forward aligner (FA) primer, which contains the Nt. BstNBI recognition site, and four bases are cleaved downstream of the recognition site. After cleavage, the fragments are separated from FA because the cleavage sequence is too short to maintain a stable Y-shape structure. Later, a linear primer (FP) containing the recognition sequence of Nt. BstNBI initiates the elongation of the polymerase to produce single-stranded DNA with the new recognition site of Nt. BstNBI. In this way, a new round of cleavage releases the FP-binding site again and initiates another round of polymerase-catalyzed chain elongation to synthesize the antisense chain (T0) of the target template. Similarly, the reverse initiation step uses T0 as the template and RA/RP is used as a primer to produce the amplified product strand T1 with the help of Nt. BstNBI and DNA polymerase. It should be noted that the researchers artificially applied the 3-terminator (e.g., 3 inverted-dT) to FP and RP to eliminate the unintended extension of the two primers along the target sequence. The first two steps not only amplify the target sequence in a linear manner, but also identify and cut the initiated strand with high specificity. Finally, the combination of T1 and FP, under the combined action of Nt. BstNBI and polymerase, leads to the formation of T2, and this, with the combination of T2 and RP, using the same method, causes the repeated production of T1 in this way, and ultimately amplifies large amounts of T1 and T2 to achieve exponential amplification. In the end, the researchers verified the product strip by running glue and used SYBR green as the signal indicator.

### 3.3. Nicking Enzymes Combined with RCA

**Figure 4:**
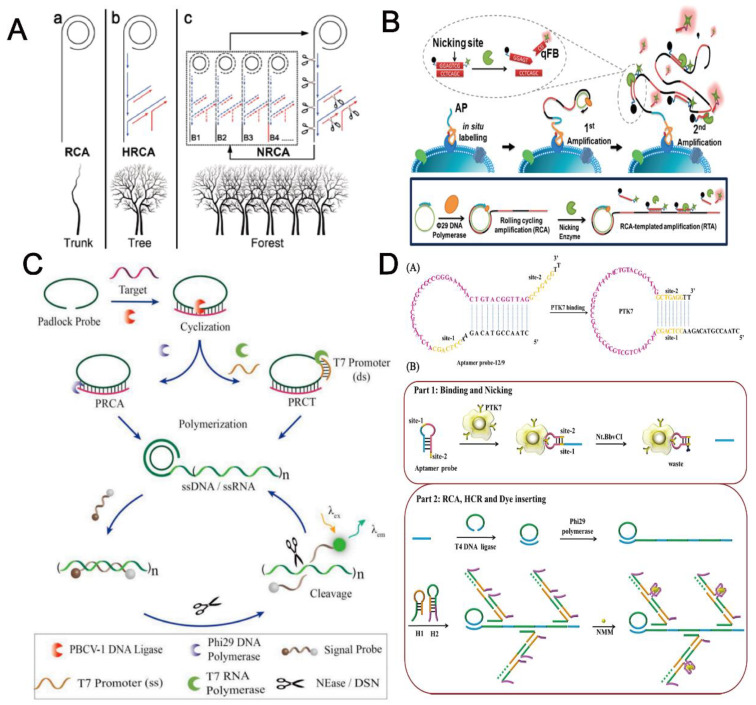
(**A**) Basic principle of nicking enzyme combined with RCA. (Reproduced with permission [74]. Copyright 2015. Royal Society of Chemistry.) RCA and nicking enzyme act together for (**B**) cancer cell phenotypic analysis. (Reprinted with permission from Ref. [75]. Copyright 2017 American Chemical Society.) (**C**) The detection of DNA or RNA. (Reprinted with permission from Ref. [76]. Copyright 2020 Elsevier.) (**D**) The analysis of living cell surface membrane proteins. (Reprinted with permission from Ref [77]. Copyright 2018 Elsevier.).

RCA, reported in the 1990s [69,70,71,72], is an isothermal nucleic acid amplification method based on ligase binding, primer extension, and strand displacement amplification reaction [73], which mimics the rolling loop replication process of microbial circular DNA in nature. Under constant temperature conditions, many repetitive sequences complementary to the ring probe can be generated. After the isothermal linear amplification of the ring probe in vitro is realized in combination with the nicking enzyme, RCA can change from linear amplification to exponential amplification or multi-primer amplification. Only a segment of a nicking enzyme needs to be designed on the ring probe. With amplification, a long strand of DNA with a specific sequence interval is obtained. By adding multiple templates and nicking enzymes, a combination of multiple templates and primers can be obtained to achieve exponential amplification (Figure 4A). Zhu et al. [74] improved HRCA to yield NRCA using nicking enzymes, which is a classic example of a nicking enzyme transforming linear amplification into exponential amplification. Herein, the authors compare RCA (linear amplification) to a tree trunk, HRCA to a tree (exponential amplification), and NRCA to a forest (exponential amplification). The HRCA tree structure was cut by the nicking enzyme into bifurcated branches, or short ssDNAs, and then each ssDNA could be used as a seed, or probe, for the growth of other HRCA trees, thus achieving signal amplification, which made an outstanding contribution to the detection of trace targets.

Based on the above principles, as shown in Figure 4B, Gao et al. [75] proposed a quantitative analysis method for plasm membrane proteins (PMPs) through an in situ rolling cycle replication template amplification strategy (isRTA) involving two rounds of amplification and a cascading isotherm reaction, which were triggered by RCA and nicking enzymes, and then a precise quantitative measurement of PMP was performed. In this work, isRTA was used to quantify tumor-associated PMP biomarkers (such as MUC1, EpCAM, and HER2) that may reflect different breast cancer phenotypes.

As shown in Figure 4C, Chuan Zhou et al. [76] designed a nicking enzyme-assisted RCA (PRCA–NESA) system, which can provide higher amplification efficiency. First, the ring probe is complementary to the target. When the target is DNA, the polymerase uses Phi29. When the target is RNA, a T7 single-stranded promoter is added and a T7 polymerase is used so that this system can be adapted for DNA/RNA amplification followed by a process by RCA with nicking enzymes; after N-segment products are amplified by RCA, the corresponding signal probe will be complementary to the product. Then, the nicking enzyme cuts the signal probe at the specified site, resulting in the release of a fluorescent signal.

As shown in 4D, to detect membrane proteins on the surface of living cells, Wei Li et al. [77] developed a nicking enzyme-assisted RCA isothermal amplification technique, in which a HCR reaction was also applied. Taking protein tyrosine kinase-7 (PTK7) as the model analyte, Wei Li et al. first designed a probe. In the absence of PTK7, site1 and site2 (two yellow parts) of the probe were separated. On the contrary, when PTK7 was present, PTK7 would complement the probe, resulting in a conformation change of the probe, and site1 and site2 would complement each other to synthesize the nicking enzyme recognition site, induce nicking enzyme cleavage, and release a triggering sequence. Then, this trigger sequence would cause an RCA response. As shown in part2 of Figure 4D, a designed circular template and T4 ligase were added to complete the RCA reaction to obtain a long sequence, which would trigger a HCR reaction with both hairpins; H2 contains the sequence producing G-quadruplex. Then, N-Methyl Mesoporphyrin IX (NMM) was added to the reaction products of HCR, and G-quadruplex was combined with MMM to generate fluorescence. In this method, a nicking enzyme was used to transform RCA and was skillfully combined with HCR, which is sufficiently sensitive to changes in PTK7 expression and provides a reliable method for the detection of living cell membrane protein expression.

### 3.4. Nicking and Extension Amplification Reaction

Nicking and extension amplification reaction, also called NEAR, was developed on the basis of SDA [78]. This technology first appeared in the form of a patent in 2009 [79], and it was continuously improved over the following years [80]. In recent years, NEAR has developed rapidly and has been put into practical applications. For example, an instrument called ID NOW produced by Abbott to detect SARS-CoV-2 virus implements the core principle of NEAR technology [81]. Compared to the SARS-CoV-2 detection platforms from Roche Cobas and Cepheid Xpert Xpress, ID now has the fastest detection speed [82].

The SARS-CoV-2 virosome of COVID-19 contains a single-stranded plus stranded RNA genome with a length of 30,000 nucleotides [83]. So the normal test involves turning SARS-CoV-2 RNA reverse transcription (RT) into complementary DNA (cDNA) and then amplifying the target region of the cDNA [84]. Studies have shown that various forms of isothermal amplification analysis have been successfully applied to detect coronavirus RNA in patient samples [85,86]. For example, Butt et al. [87] reported a dual-gene RT-lamp method for simultaneous detection of the orf1a and n genes of SARS-CoV-2, which detected 45 positive samples and 25 negative samples. The positive samples were 95% accurate, and no false-positive results or cross-reactivity were observed in the negative samples. Likewise, Yee Ling Lau et al. [88] developed and optimized a sensitive RT-RPA method for the detection of SARS-CoV-2 with a signal output utilizing SYBR Green I and/or direct lateral flow bands for rapid detection. They used 78 positive and 35 negative nasopharyngeal samples for the clinical sensitivity and specificity of RT-RPA testing. The detection limit of RT-RPA was 7.659/μL RNA and it did not cross-react with other viruses. Its clinical sensitivity and specificity were 98% and 100%, respectively. By combining EXPAR with an unprecedented reverse transcription-free (RTF) step to convert RNA to DNA, Carter et al. [89] invented an assay for SARA-COV-2 called RTF-EXPAR. The study used a special nicking enzyme called BstNI that can efficiently cut only the DNA strands of RNA–DNA heteroduplexes. This function was demonstrated by Murray et al. [90] in 2010. Carter et al. used the enzyme to generate DNA fragments and trigger the EXPAR reaction. First, they designed a 30-base DNA oligonucleotide containing a 5-base recognition site for BstNI, a sequence complementary to a portion of Orf1a of the SARS-CoV-2 RNA genome, and an EXPAR DNA template. When this DNA fragment is complementary to the viral RNA, BstNI only cleaves the DNA strand in the RNA–DNA complex and releases the short DNA as the triggering strand for EXAPR. In actual sample detection, the method accurately identified 7.25 copies/μL SARA-COV-2 RNA within 10 min. This is a good example of how research efforts can generate novel and meaningful results through the discovery and utilization of suitable nicking enzymes.

In COVID-19 testing, ID Now targets the RDRP gene of SARS-CoV-2 and provides rapid molecular results by directly testing the nasopharyngeal or oropharyngeal swabs, or by collecting the assays in fluids for detection of viral delivery agents. The detection method adopts real-time fluorescence technology. When the fluorescence intensity reaches a certain threshold, the sample can be judged to be positive, and the fastest time to reach the threshold is about 5 min. If the fluorescence threshold has not been reached at 5 min, the reaction will continue. As long as the fluorescence intensity of the amplification product reaches the threshold between 5 and 13 min, it will still be regarded as a positive sample. If the fluorescence threshold has not been reached until 13 min, it will be regarded as a negative sample [91]. The lower limit of ID NOW’s detection of COVID-19 can reach several hundred virus copies without nucleic acid extraction and the process can be completed quickly and accurately. This can be seen as an example of nicking enzymes combined with isothermal amplification for research purposes.

**Figure 5:**
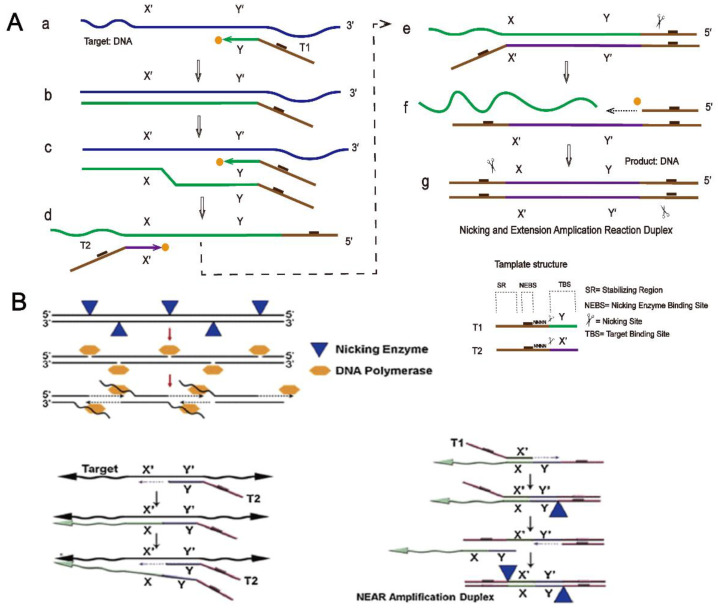
(**A**) Basic principle of nicking and extension amplification reaction. (**B**) Application of nicking and extension amplification reaction. (Reprinted with permission from Ref. [93]. Copyright 2014 Elsevier.).

The specific principle of NEAR is described in detail here. As shown in Figure 5A (a,b), template 1, which includes a stabilizing region, a nicking enzyme binding region, and a target-binding site, binds to the target, and DNA polymerase begins to extend at the 3′ end of template 1, synthesizing double-stranded DNA. (c) Template 1 binds to the target again and the polymerase begins to extend at the 3′ end of template 1 to form a new DNA single strand and replaces the old strand. (d–e) Template 2 binds to the old DNA strand that has been replaced, extends with the help of polymerase, and forms a new strand again. (e–g) The nicking enzyme cuts one of the strands and releases it. With the help of polymerase, a new strand is extended from the cutting site, thus forming DNA double strands with a nicking region binding site and a cutting site at both ends, which can double the reaction rate and increase the amplification efficiency [92].

Figure 5B shows a schematic diagram of Abbott’s Alere^TM^ i influenza A&B detection technology. The principle of this detection method is also NEAR technology. When the reaction begins, a reverse transcription step generates complementary DNA strands in viral RNA, which then serve as templates for the formation of double-stranded DNA. The next steps are similar to those shown in Figure 5A. The Alere^TM^ i influenza A&B isothermal nucleic acid amplification test is an ideal method for detecting influenza sites in children and adults because of its high sensitivity and specificity and its ability to produce results within 15 min of receiving samples [92,93].

## 4. Advantages and Disadvantages of Nicking Enzyme-Combined Isothermal Amplification Technology

With the development of molecular biology, nicking enzyme-combined isothermal amplification technology has been used to detect a wide range of analytes, including nucleic acids [94,95,96,97,98], proteins [99,100,101,102], biological small molecules [103], ions [104,105], and even cells [106]. In addition, nicking enzymes can also facilitate the development of emerging biosensors by being combined with novel bio-nanotechnologies. For example, Mengqi Huang et al. [107] established a new nucleic acid rapid localization detection method based on CRISPR/cas9 cleavage and nicking enzyme-mediated CRISPR/cas9-triggered isothermal exponential amplification reaction (Cas-EXPAR) technology. This nicking enzyme-based isothermal amplification method can achieve a detection limit of 0.82 amol with good discrimination specificity for single-base mismatches. Similarly, Shaohua Gong et al. [108] reported a DNA strand displacement amplification-assisted CRISPR-cas12a (SDACC) colorimetric assay for viral nucleic acid analysis. The SDACC strategy was able to identify single-base mismatches in the DNA sequence, enabling sensitive detection of hepatitis B virus (HBV) DNA with a minimum detection limit of 41.8 fM. Qiu Guangyu et al. [109] also proposed the establishment of a thermoplasmonic-assisted dual-mode transducing (TP-DMT) platform, which can be seen as an emerging bio-nanotechnology, with the help of a nicking enzyme to detect trace amounts of SARS-COV-2, and localized heating at the nanoscale may help enzymes work better. However, nicking enzyme-combined isothermal amplification has some disadvantages and advantages. As shown in Table 2, we compare some of the amplification techniques mentioned in this review.

EXPAR requires fewer enzymes in the amplification process [60], so it is more promising in POCT, and a short strand of DNA or RNA can act as a trigger sequence to trigger a reaction [66]. Although LAMP or NEAR performed well in the amplification and detection of large nucleic acids, they were not suitable for short miRNAs. Therefore, EXPAR is competent for the detection of miRNAs. However, this method has certain limitations. For EXPAR, reaction temperature is not only an important parameter determining polymerase and enzyme activities but also an important factor determining amplification efficiency and specificity. For example, at high temperatures of 55–60 °C, it is suitable for DNA polymerase with strong strand displacement activity, which can induce high amplification efficiency. High temperatures can also reduce non-specific DNA interactions. However, for some applications, such as those involving antibody and protein analysis, a lower temperature may be required for the binding of antibodies to target proteins at 37 ° C. While the lower temperature increases the stability between the target (or trigger DNA) and the template, it may lead to more non-specific DNA interactions and higher background signals. From what has been discussed above, high background magnification is a major challenge for EXPAR, limiting its practical application.

In terms of the original SDA and RCA, their disadvantages are obvious; they are both linear amplification methods and amplification speed is slow. Thanks to the nicking enzyme, linear RCA and linear SDA were transformed into exponential RCA and exponential SDA. Although exponential amplification techniques offer high amplification efficiency and detection sensitivity, they suffer from rapid non-specific amplification and complex designs. In contrast, the linear amplification strategy is more convenient and excludes the interference of non-specific amplification. Unlike E-SDA, which has bidirectional amplification properties, the amplification of the original SDA is unidirectional. This means it has a simple design. Although the detection sensitivity is low, it does not affect its application [110]. However, with the original SDA, there is a risk that the restriction enzyme will cut both strands of DNA. It is worth noting that although SDA occurs at a constant temperature in the amplification process, the formation of primer–template complexes at the beginning requires a heating process (95 °C) [108]. The reaction temperature of RCA (~37 °C) is lower than that of HRCA (~60 °C), so the RCA technique can be used for in situ analysis of living cells [111]. Although linear RCA amplification is slow, it can be improved by adding ssDNA-binding proteins. Since the product of RCA is ssDNA in the shape of a thread that repeats the target sequence, in this linear format it was transformed into E-RCA after adding a nicking enzyme, and there is an advantage in that adding a second primer to E-RCA is unnecessary. Finally, both types of RCA products can be verified by gel.

To better illustrate the advantages and disadvantages of NEAR, here, the ID Now technology developed by Abbott based on the NEAR principle is taken as an example. The technology was originally designed to detect flu A&B, Streptococcus B, and respiratory syncytial virus [81,112]. Previous comparative studies have shown that ID Now, with significant improvements in both amplification and analysis software [113,114], has a sensitivity of 93.2%/97.2% for detection of influenza A/B, with specificity greater than 97% compared to the RT-PCR assay platform; the ID Now assay platform had the lowest inefficiency (0.5%) and the time to obtain positive samples was shorter [112,115]. The disadvantage is that, while it performs well in strong and moderate positive samples, it is less sensitive to weak positive samples [116]. Another major limitation of this approach is that the technique can only test one sample at a time and trained personnel are required to carry out the operation.

As a result of this shortcoming, in the context of the current COVID-19 pandemic, many experimental methods still rely on traditional high-throughput qPCR batch tests. Moreover, the specificity and sensitivity of NEAR technology depends on specially designed templates and nicking enzymes and the templates are relatively difficult to design.

Finally, there is a common shortcoming. In terms of detection cost, the cost of nicking enzymes for isothermal amplification is generally higher than that of traditional real-time fluorescent PCR enzymes, so the detection cost of nicking enzyme-combined isothermal amplification technology is higher than that of qPCR. However, despite the complexities and difficulties of isothermal amplification, the system, in terms of its design, compatibility, and application, is more intuitive and easy to use. Using the specific reaction temperature required for nicking enzymes, it is expected to be easily deployed in more settings, especially in poor mountainous areas [118], and the reaction may be able to be carried out in warm water.

## 5. Conclusions and Prospects

The limitations of traditional PCR methods have become increasingly obvious [122]. Although isothermal amplification has some advantages over PCR [123], many clinical practices and past research experience tell us that isothermal amplification has a lot of room for improvement. On this basis, nicking enzymes, modified and combined with several isothermal amplification techniques, play a central role in many fields. This review has focused on different types of conditions and processes for nicking enzyme-combined isothermal amplification, its advantages and disadvantages compared to unmodified nucleic acid amplification methods, and its application in nucleic acid analysis, immunoassays, and other molecular detection methods.

In terms of application prospects, nicking enzymes can identify specific sites but only for cutting single strands of double-stranded nucleic acids. Taking advantage of this characteristic, in conjunction with isothermal amplification, we can search for a specific target to build many isothermal amplification sensing platforms [124,125] (electrochemical platform, optical platform, etc.). In terms of signal output, whether directly used for detection or signal amplification, or in combination with other detection platforms, nicking enzyme-combined isothermal amplification analysis can play a positive role. In addition to its important role in detection, many studies have also reported that this reaction can optimize oligonucleotide short strand synthesis [126,127] for use in testing the abilities of polar molecules, etc. [128]. Moreover, the nicking enzyme also plays an indispensable role in using nucleic acids to build dynamic models [129,130,131,132]. However, nicking enzyme-combined isothermal amplification technology has certain limitations in terms of applications. First, in intracellular and in vivo assays (e.g., intra-cellular miRNA imaging), enzymatic-free amplification is often used, which can provide good signal output. Nicking enzyme-combined isothermal amplification strategies are difficult to implement in cells or in vivo, and current solutions are to directly use intra-cellular enzymes or transfer enzymes into cells. We also expect more suitable nicking enzymes to be discovered or to be retrofitted to solve this problem. Second, our analysis of cellular non-nucleic acid biomolecules (such as protein–protein interactions) often requires additional components to be inputted into the cell, such as the use of DNA/RNA-based nucleic acid aptamers or azide-incubated cells for anchoring DNA. How to improve and simplify the application of nicking enzyme-combined isothermal amplification technology in vivo or in cells are questions worthy of consideration. The bottom line is that these amplification methods have not been well commercialized or developed for clinical diagnostics, and handheld devices are critical for the POCT scenario. In general, we can be sure that there is huge room for development of nicking enzyme-combined isothermal amplification technology. We also expect that more and more nicking enzymes can be discovered and the technology continuously enhanced to improve performance so that it will be able to be applied and developed in more scenarios and fields.

## Figures and Tables

**Table 1:** Characteristics of important nicking enzymes.

Enzyme	Recognition Sequence	Isolation Source	Ref
Nb. BbvCI	3′-GGAGT˄CG-5′	*Bacillus brevis* strain C	[28,31]
Nb. BsmI	3′-CTTAC˄GN- 5′	*Bacillus stearothermophilus* NUB 36	[32,33]
Nt. BstNBI	5′-GAGTCNNNN˅N-3′	*Bacillus stearothermophilus*	[33,34,35]
Nt. BspQI	5′-GCTCTTCN˅N-3′	*Bacillus* sp. strain Q	[28,36]
Nt. BspD6I	5′-GAGTCNNNN˅N-3′	*Bacillus* sp. strain D6	[37]
Nt. Bst9I	5′-GAGTCNNNN˅N-3′	*Bacillus stearothermophilus* strain 9	[32]
Nt. BstSEI	5′-GAGTCNNNN˅N-3′	*Bacillus stearothermophilus*	[33]
Nt. BsmAI	5′-GTCTCN˅N-3′	*Bacillus stearothermophilus* A664	[33]
Nt. AlwI	5′-GGATCNNNN˅N-3′	Engineered endonuclease	[38]
Nb. BsrDI Nt. CviPII Nt. CviQII	5′-GCAATG˄NN-3′ 5′-C˄CD-3′ 5′-R˄AG-3′	*Bacillus stearothermophilus* D70 *Chlorella* virus NYs-1 *Chlorella* virus NY-2A	[39] [33] [29]

(Note: D = A or G or T, R = A or G, N = A or G or C or T; “˄” indicates that the complementary strand of the sequence is recognized and cut; “˅” indicates that the sequence itself is recognized and cut.).

**Table 2:** Comparison of various isothermal amplifications.

Parameters	PCR	Linear SDA	Linear RCA	EXPAR	E-SDA	E-RCA	Ref
Enzymes (Numbers and names)	1, Polymerase		1, Polymerase	2: Polymerase and NEase	2: Polymerase and NEase	2: Polymerase and NEase	[3,6,13,52,105,117,118,119,120,121]
Primer	2	1	1	0	2 or 4	≥2	
Temperature (°C)	Varies between 4–95	~37	~37	∼60	~37	~60	
Reaction time(h)	>2	>1	>1	~0.5	1–2	1–4	
Target	DNA	DNA	RNA (DNA)	short DNA (RNA)	DNA	RNA (DNA)	
Amplification	DAN	DNA	RNA (DNA)	RNA (DNA)	DNA	RNA (DNA)	
Efficiency	2^n^	<10^7^	10^3^	10^6^–10^9^	10^7^	10^7^	

(Note: n = number of cycles.).

## Data Availability

Not applicable.
